# Wheat Selenium-binding protein TaSBP-A enhances cadmium tolerance by decreasing free Cd^2+^ and alleviating the oxidative damage and photosynthesis impairment

**DOI:** 10.3389/fpls.2023.1103241

**Published:** 2023-02-07

**Authors:** Fei Luo, Dong Zhu, Haocheng Sun, Rong Zou, Wenjing Duan, Junxian Liu, Yueming Yan

**Affiliations:** Beijing Key Laboratory of Plant Gene Resources and Biotechnology for Carbon Reduction and Environmental Improvement, College of Life Science, Capital Normal University, Beijing, China

**Keywords:** wheat, Selenium-binding protein, Cd tolerance, oxidative stress, photosynthesis impairment, CXXC motif

## Abstract

Cadmium, one of the toxic heavy metals, robustly impact crop growth and development and food safety. In this study, the mechanisms of wheat (*Triticum aestivum* L.) selenium-binding protein-A (TaSBP-A) involved in response to Cd stress was fully investigated by overexpression in Arabidopsis and wheat. As a cytoplasm protein, TaSBP-A showed a high expression in plant roots and its expression levels were highly induced by Cd treatment. The overexpression of *TaSBP-A* enhanced Cd-toleration in yeast, Arabidopsis and wheat. Meanwhile, transgenic Arabidopsis under Cd stress showed a lower H_2_O_2_ and malondialdehyde content and a higher photochemical efficiency in the leaf and a reduction of free Cd^2+^ in the root. Transgenic wheat seedlings of TaSBP exhibited an increment of Cd content in the root, and a reduction Cd content in the leaf under Cd^2+^ stress. Cd^2+^ binding assay combined with a thermodynamics survey and secondary structure analysis indicated that the unique CXXC motif in TaSBP was a major Cd-binding site participating in the Cd detoxification. These results suggested that TaSBP-A can enhance the sequestration of free Cd^2+^ in root and inhibit the Cd transfer from root to leaf, ultimately conferring plant Cd-tolerance *via* alleviating the oxidative stress and photosynthesis impairment triggered by Cd stress.

## Introduction

As an allohexaploid species, wheat (*Triticum aestivum* L., 2n=6x=42, AABBDD) has a wide adaptability and provides a stable source of carbohydrates and proteins duo to its uncommon genetic potential that synchronizes its flowering time with diverse environmental conditions (Kamran et al., 2014). Wheat consumption is globally estimated to rise 70% in the next few decades (2020–2050) as the human population increases (Vitale et al., 2020). However, many anthropogenic industrial activities including electroplating, mining, battery production and iron and steel plants as well as widely used pesticides and phosphate fertilizers in modern agriculture are some of major sources of heavy metal such as cadmium (Cd), chromium (Cr), mercury (Hg), lead (Pb), copper (Cu), zinc (Zn) and nickel (Ni) to soils (Choppala et al., 2013; Haider et al., 2021; Qin et al., 2021). Those increasingly deteriorative contaminations in agricultural soils have become a serious threat to grain production worldwide (Rizwan et al., 2012; Caparrós et al, 2022). It is estimated that 12 million tons of grain are polluted each year by heavy metals in China (Qin et al., 2021). Among of those heavy metal, the accumulation of Cd was continuously increasing, while others was gradually decreasing during the period from 2005 to 2017 (Huang et al., 2019). Cd generally poses high toxicity to both plants that and humans, even at a very low concentration (López-Luna et al., 2015). The accumulation of Cd between 5–10 mg Cd kg^-1^ (dry matter) in plant tissue is toxic to most plants (Choppala et al., 2014). For human, the maximum dietary exposure of Cd is 25 μg kg^-1^ body weight per month according to FAO/WHO. Humans often contact with Cd-polluted foods *via* the food chain (Dai et al., 2012). When exposed to Cd contaminated environments, people would have a high risk of acquiring many diseases, including chronic kidney disease, osteoporosis, cardiovascular diseases, and cancer (Fatima et al., 2019). Therefore, it is significant to ensure food safety and human health to pay attention to heavy metal pollution, especially Cd- contaminations.

Cd stress has significant effects on plant growth and development at both morphological and physiological levels (Shanying et al., 2017). In morphological level, Cd toxicity was reported to cause a substantial decrease in total leaf area, root length and tips, and dry weight of plant leaves, stems and roots (Jinadasa et al., 2016; Rizwan et al., 2017). In physiological level, Cd stress causes damages in photosynthetic apparatus and Calvin cycle related enzymes, resulting in a decline of photosynthesis and carbon assimilation rate (Pietrini et al., 2010; Ying et al., 2010). Furthermore, Cd stress can induce water stress, leading to decreases in stomatal conductance, transpiration rate, and relative-water content of plant leaves (Arasimowicz-Jelonek et al., 2011; Najeeb et al., 2011). The signal transduction induced by Cd generally triggers reactive oxygen species (ROS) production, which damages cellular organelles and biomolecules (Ranieri et al., 2005; Gratão et al., 2015). In addition, Cd stress has negative effects on plant mineral content by interfering with the intake of mineral nutrients such as zinc (Zn), iron (Fe), calcium (Ca), manganese (Mn), magnesium (Mg), copper (Cu), silicon (Si), and potassium (K) (Khan et al., 2015a; Jinadasa et al., 2016). In summary, the physiological disorders caused by excessive intake of Cd^2+^ under Cd stress often produce a severe inhibition of morphology and grain yield loss in agriculture (Li et al., 2015).

Plants have evolved different mechanisms to resist Cd stress during the long evolutionary process, including restricting metal uptake and enhancing their detoxification abilities (Choppala et al., 2014; Wei et al., 2022). When plants subject to heavy metal stress in the soil solution, the cell walls serve as the first barrier against metal toxicity (Lang and Wernitznig, 2011). The cell wall of the stems, leaves and fruits was reported to involve in Cd immobilization in bush beans and pepper (Xin and Huang, 2014; Xin et al., 2014). Once Cd enters the cytosol, plants can form metal chelates/complexes to minimize the concentration of free Cd^2+^ in the cytosol (Sarwar et al., 2010; Saraswat and Rai, 2011). So far, two principal peptides have been found to participate in chelating to Cd: phytochelatins and metallothioneins (Ismael et al., 2019). The thiol moieties of phytochelatins and cysteine-rich small polypeptides can chelate metal ions, including Cd, Cu, Zn and Ag etc. (Cobbett and Goldsbrough, 2002; Uraguchi et al., 2017). Meanwhile, metallothioneins belonging to cysteine-rich proteins with a low molecular mass can resist to Cu and Cd stresses (Jianmin and Goldsbrough, 1994). Additionally, the vacuoles/plastid sequestration and metal efflux mechanism also significantly contributes to enhance plant detoxification abilities to Cd stress (Ogawa et al., 2009; Wang et al., 2015). It is known that some metal transporters can mediate these processes, such as CAX2/4 (Korenkov et al., 2007), HMA1/3 (Lei et al., 2020; Zhang et al., 2020), MRP3 (Verbruggen et al., 2009), ABCC3/9/13 (Bhati et al., 2016; Yang et al., 2021), and metal efflux transporters PCR1/2 (Lin et al., 2020), PDR8 (Kim et al., 2007), and MATE (Li et al., 2002; Wang et al., 2015).

The transcriptional regulation is an important strategy for plant heavy metal stress response. To date, many Cd-responsive transcription factors in plants have been identified and characterized, such as Hsfs (Shim et al., 2009; Chen et al., 2020), ERFs (Lin et al., 2017), ORG3 (Xu et al., 2017), WRKYs (Cai et al., 2020), MYBs (Agarwal et al., 2020), and bHLHs (Yao et al., 2018), etc. As the key downstream effectors of Cd stress transcriptional pathways, these Cd-responsive transcription factors can trigger the expression of Cd-detoxification genes and converge Cd stress signals (Chmielowska-Bąk et al., 2014). In addition, a lot of metallochaperones can traffic metal ions in cytosol. Saccharomyces cerevisiae metal homeostasis factor (ATX1) can bind a single Cu ion by two cysteines in the MXCXXC motif (here M, X and C represents methionine, any amino acid and cysteine, respectively) (Lin and Culotta, 1995; Rousselot-Pailley et al., 2006). This motif is also present in numerous metal binding proteins, such as the P-type copper transporter CCC2 (Yuan et al., 1995), bacterial carriers for mercury ions MerP (Powlowski and Sahlman, 1999), copper chaperones for SOD1 CCS (Culotta et al., 1997), and the cadmium binding protein Cd19 (Suzuki et al., 2002).

Wheat, compared to other cereals such as maize and rice, can accumulate more Cd mainly *via* the roots and then transfer it from roots to aerial parts, contributing to enrichments in the grain eventually (Greger and Löfstedt, 2004; Jafarnejadi et al., 2011). Therefore, it is significant to understand the mechanism of wheat response to Cd stress, which may be managed to alleviate Cd uptake or accumulation, promoting wheat growth and improving grain yield and quality. During the past decades, a number of strategies, such as the selection of low Cd-accumulating wheat cultivars (Liu et al., 2020), exogenous application of plant growth regulators (Agami and Mohamed, 2013; Wang et al., 2017b), the use of inorganic amendments (Khan et al., 2015b; Naeem et al., 2018; Cheng et al., 2021; Ma et al., 2022), organic amendments, nanoparticles (Grüter et al., 2019) have been applied for the alleviation of Cd toxicity in wheat (Zhou and Li, 2022). Low-Cd or high Cd-resistance wheat cultivars were increasingly fostered with molecular genetics and breeding approaches developed rapidly. (Zaid et al., 2018). Cd tolerance was enhanced in rice expressing *TaHsfA4a* by upregulating metallothionein gene expression (Shim et al., 2009). The *OsHMA*3 overexpression highly inhibited Cd accumulation in wheat grain by decreasing root-to-shoot Cd translocation nearly 10-fold (Zhang et al., 2020). The overexpression of durum wheat TdSHN1 conferred Cd resistance by promoting the activities of superoxide dismutase (SOD) and catalases (Djemal and Khoudi, 2022).

The candidate genes related to enhance crop tolerance to heavy metals are urgently needed to ensure food safety. The selenium-binding protein (SBP) is a typical SBP56 family member, which was identified in *A. thaliana* (Dutilleul et al., 2008; Hugouvieux et al., 2009; Schild et al., 2014). Early studies identified SBP as a cytosolic selenium binding protein, named as SBP56 in mouse liver, which was found to bind selenium (Bansal et al., 1989; Bansal et al., 1990). It is involved in intra-Golgi protein transport in Mammalia (Porat et al., 2000), and the decreased levels of SBP1 are associated with epithelial cancers and breast cancer (Yang and Diamond, 2013). Selenium-binding protein in plants, first found in *Lotus japonicas*, participated in nodule formation during the symbiosis of plants and rhizobia (Flemetakis et al., 2002). AtSBP1 has been reported that it can interact with glutaredoxins AtGRXS14 and AtGRXS16 that contain a PICOT domain and belong to part of the plant’s response to oxidative stress (Valassakis et al., 2019). It also served as an interacting partner of DAD1-LIKE LIPASE 3 (DALL3) that participated in the network of genes regulated by cadmium (Dervisi et al., 2020). The overexpression of *SBP1* in rice could improve plant tolerance to different pathogens (Sawada et al., 2004). In particular, overexpressing *AtSBP1* in Arabidopsis enhanced tolerance to Cd stress in both suspension cells (Sarry et al., 2006) and entire plants (Dutilleul et al., 2008). Meanwhile, *AtSBP1* can also participate in Zn, Cu and H_2_O_2_ stress responses (Hugouvieux et al., 2009). The Se-binding site Cys^21^Cys^22^ in AtSBP1 was identified, which could form SeCys [R-S-Se(II)-S-R] and confirmed Se tolerance (Schild et al., 2014). The overexpression of *AtSBP1* could produce greater Cd accumulation in Arabidopsis (Dutilleul et al., 2008), indicating that AtSBP1 could enhance the Cd uptake. However, the state (free or complex) of the accumulated Cd in the overexpressed plants remains unclear; this is critical as it will allow researchers to further understand the mechanisms of SBP for Cd tolerance.

In the current study, a comprehensive investigation was performed to reveal the molecular mechanisms of wheat TaSBP-A enhancing Cd tolerance. We focused on dissecting the detoxification function of TaSBP-A *via* a specific Cd-binding motif. Our purpose is to provide new insights into the Cd-tolerant mechanisms of plants, which could be beneficial for improving the Cd tolerance as well as reducing grain Cd accumulation of crop cultivars.

## Materials and methods

### Wheat materials, seedling cultivation and Cd treatment

Common wheat variety Chinese Spring (CS) was used as material, and the mature seeds were cultivated based on the previous report (Zhang et al., 2014). In brief, the seedings were cultivated in Hoagland solution. The Cd stress treatment at three-leaf stage was conducted using 50 µM CdCl_2_ in Hoagland solution. Three biological replicates were set in both treatment and control, and the samples of roots, stems (crown to ligule) and leaves were respectively collected from 24, 48, 72 h treatments and control, and then immediately immerged into liquid nitrogen prior to use.

### RNA-seq and RT-qPCR

The expression profiling of *TaSBP* genes in different organs including roots, stem axis, leaves was detected using the RNA-seq database of wheat (http://www.wheat-expression.com/genes/heatmap?gene_set=RefSeq1.1&genes=TraesCS3A02G422100%2CTraesCS3D02G417500%2CTraesCS3B02G457600). Total RNA isolation, cDNA synthesis and real-time quantitative polymerase chain reaction (RT-qPCR) were based on the previous report (Yu et al., 2016). The specific primers were shown in **Table S3**. The expression levels of *TaSBP* were presented as values relative to the corresponding control samples at the indicated times and conditions after normalization to *Ubqutin* (UBI) transcript levels.

### Subcellular localization

The *TaSBP-A* gene clone, vector (16318) construction and leaf protoplast transformation of Chinese Spring were based on previous report (Zou et al., 2020). The specific primers were shown in **Table S3**. Confocal laser scanning microscope (Leica TCS SP5, Germany) was used for monitoring the GFP signal and chlorophyll red auto-fluorescence.

### Overexpression of TaSBP-A in *Saccharomyces cerevisiae*

*Saccharomyces cerevisiae* strain DEY1457 (*MATα can1 his3 leu2 trp1 ura3 ade6*), kindly provided by Prof. Liping Yin, Capital Normal University, was used for heterologous expression of TaSBP-A protein. The full gene CDS of TaSBP-A was cloned in the destination vector pYES2 (Invitrogen). The specific primers were shown in **Table S3**. The empty vectors (EV) and combined vectors were respectively transformed into yeast strains DEY1457 following standard procedure (Invitrogen). Single colonies cultured in the exponential phase (OD 2.0) were diluted to six stepped concentration (OD 2, 2 x 10^-1^, 2 x 10^-2^, 2 x 10^-3^, 2 x 10^-4^, 2 x 10^-5^) and drop on the dextrose-Ura solid medium with/without 30 μM Cd^2+^. Three independent clones were used for the experiments.

### Overexpression of TaSBP-A in Arabidopsis and wheat and Cd stress treatment

Arabidopsis genetic transformation was based on previous report (Li et al., 2017). pCAMBIA1302 with a 3×Flag-tag was used as expression vector, and at least two generations of resistance screening were carried out. All plants were grown in a growing chamber at 21–22°C with cool-white fluorescent light (80–100 µmol m^−2^s^−1^) in a long day photoperiod (16 h light/8 h dark). Arabidopsis Seedlings were cultivated for 7 days in half-strength Murashige and Skoog (1/2 MS medium) supplemented with 0.6% (w/v) sucrose and 0.7% (w/v) agar, and then transferred to 1/2 MS medium with 0, 75, 150 μM CdCl_2_ for another 7 days in growth chamber. The measurement of seedling root length and fresh weight and statistics of survive rate were performed after 7 days treatment in 1/2 MS medium. At the same time, one-week old Arabidopsis plants were cultivated in garden soil (Basic substrate No. 1, Pindstrup Mosebrug A/S, Denmark) without additional fertilizer for Cd stress treatment. Young plants were grown for three weeks in garden soil, and then used for Cd treatment by watering with and without 150 μM CdCl_2_ solution, respectively.

Full length of *TaSBP-A* CSD was cloned into wheat expression vector, pWMB110, with a HA tag under the control of maize *UBI* promoter. The new vector was transformed into *Agrobacterium tumefaciens* strain C58C1 by triparental mating, and then further introduced into W48 immature embryos to generate transgenic plants according to the methods described by previous report (Wang et al., 2017a). The mature seeds from three independent stable transgenic lines at T3 generation were geminated in filter paper soaked with distilled water. After 48 h, uniformly germinated seeds were selected to grow in the half strength Hoagland’s nutrient solution. Cd stress treatment was applied to W48 (non-transgenic control) and *TaSBP-A* overexpressed wheat seedlings at three-leaf stage with 0 and 50 μM CdCl_2_ for two weeks. The measurement of the fresh weight and root length were performed after 2 weeks treatment. The seedling leaves and roots were respectively collected for the measurement of Cd content.

### Measurements of chlorophyll, malondialdehyde and H_2_O_2_ content, SOD activity and chlorophyll fluorescence

Wild-type and transgenic Arabidopsis plants were treated with 150 μM Cd for 4 weeks. The measurement of chlorophyll and malondialdehyde (MDA) content and the detection of superoxide dismutase (SOD) activity were carried out according to previous report (Li et al., 2017). H_2_O_2_ content was measured using kit (KGT018, KeyGen Biotech, China) based on the manufacturer’s instructions. Chlorophyll fluorescence was detected by using IMAGING‐PAM chlorophyll fluorometer (Walz, Effeltrich, Germany) as previous report (Schreiber et al., 2007). The wild‐type and transgenic plants were treated by 20 photons m^-2^•s^-1^ (actinic light) after dark-adaptation. And then maximal PSII quantum yield (F_v_/F_m_) was measured. The equation was used to calculate F_v_/F_m_ : F_v_/F_m_ = (F_m_–F_0_)/F_m_. The effective PSII quantum yield (ΦPSII) was determined by using the formula: ΦPSII = (F_m_′-F)/F_m_′. The inhibition of PSII quantum yield (Inh) was detected by using the equation: Inh = (ΦPSII control–ΦPSII sample)/ΦPSII control.

### Measurement of total Cd content in yeast cells and plant extracts

Cd-treated and untreated yeast cells and plant leaves were washed with Cd^2+^ free medium or ddH_2_O, and dried for 3 d at 55°C, and then put into digestion tank. Pre-digesting was conducted by adding 5 mL 65% HNO_3_ (Suprapur; Merck) and 2 mL H_2_O_2_ (Suprapur; Merck) to the digestion tank for 40 min at room temperature. The samples were digested by microwave digestion instrument (MARS, CEM Corporation, USA) for 2 h. Cd content (ng/g DW) was calculated using inductively couple mass spectrometry (ICP-MS, ELAN DRC-e, PerkinElmer) according to previous report (Maher et al., 2001).

### Measurement of Cd^2+^ fluxes and Cd microscopic imaging

Cd^2+^ fluxes from one-week-old Arabidopsis seedlings cultivated on 1/2 MS were detected. Net fluxes of Cd^2+^ in root hair were measured by the noninvasive micro-test technique (NMT; BIO-001A, Younger United States Science and Technology Corp, Beijing, China) combined with IFLUXES/IMFLUXES 2.0 software (NMT100 Series, Younger USA, Amherst, MA, USA) (Ma et al., 2015). The microelectrodes were calibrated in 0.1 and 0.01 mM Cd^2+^ before the measurements of Cd^2+^ flux. The electrodes with Nernstian slopes were > 29 ± 3 mv/decade. Arabidopsis seedlings were transferred into a measuring chamber containing 10 mL of measuring solution (0.1 mM KCl, 0.03 mM CdCl_2_, and 0.3 mM MES, pH 5.8.) and equilibrated for 10 min before measurement.

Visualization of free Cd^2+^ in Arabidopsis roots was conducted in one-week-old seedlings. The Cd probe Leadmium™ Green AM dye (Molecular Probes, Invitrogen, Calsbad, CA, USA) was utilized to detect the distribution of Cd in plant roots pre-treated with 150 μM Cd^2+^ for 0, 6 and 12 h. Cd fluorescence was excited at 488 nm and visualized using Zeiss LSM 780 (Carl Zeiss, Germany).

### Overexpression of TaSBP-A/ΔTaSBP in *E. coli* and purification of the recombinant proteins

The mutant (ΔTaSBP-A) with displaced Cys with Gly in the putative metal binding regions was constructed. cDNA in the entry clone was cloned into the destination vectors pEGX-4T-1, used to produce GST-TaSBP-A/ΔTaSBP protein carrying additional 250 amino acids at the N terminus compared to the recombinant proteins. The recombinant plasmids were transformed into *E. coli* strain BL21. The cell cultures were fostered at 37°C for 3-4 h until the OD = 0.6-0.8, and then 1 mM IPTG was added for induction 16 h at 16°C. The recombinant proteins were isolated by batch purification with glutathione sepharose 4B according to the manufacturer’s instructions (Amersham). Protein concentrations were determined by spectrophotometer (NanoDrop 2000, Thermoscientific) at 280 nm.

### *In vitro* Cd^2+^ binding shift and binding ratio measurement

Two constructs wild type with the normal CXXC motif (TaSBP-A) and mutant with the GXXG motif (ΔTaSBP-A) were prepared. The recombinant protein (100 μM) of TaSBP-A/ΔTaSBP was incubated with 500 μM CdCl_2_ and 500 μM CdCl_2_ together with 500 μM EDTA at 4°C overnight. The shift was tested by using 10% SDS-PAGE. The recombinant plasmid of pEGX-*TaSBP-A/ΔTaSBP* was transformed into *E. coli* BL21 (DE3) and induced at 16°C for 10 h with 1 mM IPTG. Then, 100 μM CdCl_2_ was added to the liquid medium for 10 h culture. The cells were collected and mildly lysed using BugBuster Master Mix (Novagen, 71456-4). The lysis was centrifuged at 16000 g and 4°C for 15 min. The purified proteins were divided into equal two parts: one used for measuring Cd content by ICP-MS, and the other for measuring the protein concentration by DC protein determination kit (Bio-Rad). The stoichiometry of protein binding to Cd was conducted according to Cd and protein concentration (Luo et al., 2019).

### Thermodynamic parameters determined by isothermal titration calorimetry and secondary structure characterization by circular dichroism

Cd-TaSBP-A/ΔTaSBP thermodynamic parameters were detected by calorimetric experiments of the recombinant TaSBP-A/ΔTaSBP proteins. Calorimetric titrations were conducted at 25°C with stirring at 1000 rpm with a filter time constant of 2 s by using a microcalorimeter (Microcal ITC 200 System, GE Healthcare). Negative controls were performed by the injections into the buffer, resulting only in signals from heat of salt dilution.

Secondary structure characterization (modifications) of the recombinant TaSBP-A protein after incubation with Cd^2+^ was performed by circular dichroism (CD). The recombinant protein of TaSBP-A/ΔTaSBP (1 nmol) was incubated with gradient concentration of Cd^2+^ (0, 2, 3, 4 nmol) in 250 μL incubation buffer. Spectra acquisition at 25°C used a spectropolarimeter (J-815, Jasco) at the far UV (200–260 nm). The following parameters were set: 1 nm step, 2 nm band width, and scan speed 200 nm/min with the optical path length 1 mm. The assessment of protein second structure was performed by K2D method based on the linear regression method (Yang et al., 1986).

### Genetic transformation of TaSBP-A and ΔTaSBP-A in wheat protoplasts and viability comparison under Cd treatment

The wheat protoplasts were cultivated overnight after transformation of plasmids including 16318hGFP, reconstructive *TaSBP-A* and *ΔTaSBP-A*, respectively. Then, the viable protoplasts were treated with 50 μM CdCl_2_ for 0, 2, 4, 6, 8, 10, and 12 h. To determine cell viability, the GFP fluorescence and chloroplast autofluorescence were observed by confocal laser scanning microscope (Leica TCS SP5, Germany). The transgenic protoplasts were cultivated in 0 μM CdCl_2_ as the control (CK). The relative rate of protoplasts viability (treatment group/CK) was counted from 0 to 12 h.

## Results

### Phylogenetics and structural characterization of TaSBPs

**Figure 1:**
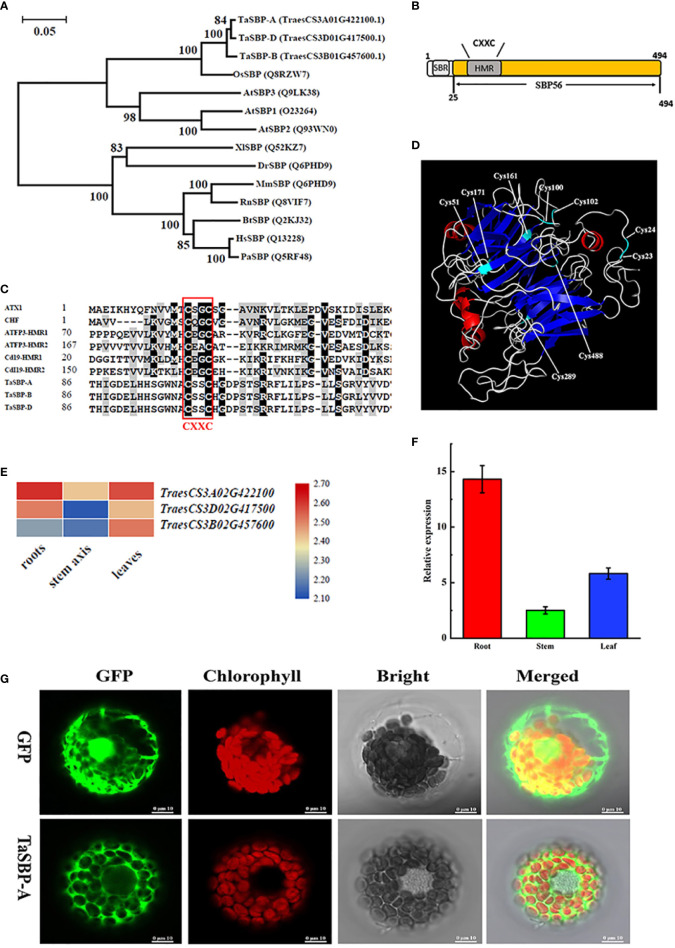
Phylogenetics and structural characterization of wheat TaSBPs. **(A)** Phylogenetic relationships between TaSBP-A/B/D protein and other SBPs of various species. **(B)** Simplified illustration of TaSBP protein structure. One putative metal binding region (HMR) and one Selenium-binding region (SBR) are shown. **(C)** Protein sequences containing the CXXC-type metal binding domain were acquired from GenBank. Accession numbers include ATX1 (P38636), CHF (AAC33510), ATFP3 (AAD09507) and Cd19 (AAM64219.1). The core sequence of the metal binding region is CXXC (here C represents cysteine and X represents any amino acid). **(D)** 3-D structure of TaSBP-A based on the structure of *S. tokodaii* (SMTL ID: 2ece.1). **(E)** The heat map of *TaSBP’s* expression in the root, stem and leaf of wheat seedings. **(F)** The expression of *TaSBP-A* in different organs of *Chinese Spring* at the three-leaf stage. **(G)** Subcellular distribution of the 35S-TaSBP-GFP fusion proteins in wheat protoplasts as shown by confocal laser scanning microscope. The red represents the chloroplast fluorescence and the green indicates the GFP fluorescence.

Three protein sequences (TraesCS3A01G422100.1, TraesCS3B01G457600.1 and TraesCS3D01G417500.1) in Chinese Spring Protein database (http://plants.ensembl.org/index.html) were homologous to Arabidopsis SBPs (AtSBP1/2/3); in turn, they respectively located on chromosomes 3A, 3B and 3D. Specifically, TraesCS3A01G422100.1 showed 98.59% and 97.37% identities to TraesCS3B01G457600.1 and TraesCS3D01G417500.1, respectively (**Figure S1**). This demonstrates that hexaploid wheat had only three SBP copies, and they are named TaSBP-A, TaSBP-B and TaSBP-D. A phylogenetic tree constructed by 11 SBPs from different animal and plant species showed that TaSBP-A/B/D had a close phylogenetic relationship with the SBP56 family (**Figure 1A**).

Structural characterization showed that TaSBPs belonged to the SBP56 family and contained a putative heavy metal binding motif as well as a selenium-binding site CXXC (**Figure 1B**). They also showed a partial similarity with other typical metal binding proteins in the metal binding region CXXC (**Figure 1C**). A three-dimensional model analysis of TaSBP-A was performed by using SWISS-MODEL (https://swissmodel.expasy.org/) to identify the potential Cys residues involved in Cd binding. As the closest homologue, the structure of the hypothetical selenium-binding protein from *Sulfolobus tokodaii* (SMTL ID: 2ece.1) was used to generate a three-dimensional model with the program Modeler (Global Model Quality Estimation of 0.7; 43.91% sequence identity). As shown in **Figure 1D**, nine potential Cys residues were located on the surface of the TaSBP-A protein, among which four Cys residues, Cys^100^, Cys^102^, Cys^161^, and Cys^488^, showed high conservation. Cys^23^ and Cys^24^ were conserved in all the photosynthetic organisms, which were identified as the selenium-binding sites in Arabidopsis (Schild et al., 2014). The Cys^100^ and Cys^102^ on the random coil belonged to a CXXC motif related to metal binding (Lin and Culotta, 1995; Yuan et al., 1995; Culotta et al., 1997).

### Expression and subcellular localization of TaSBP-A

RNA-seq analysis of the three *TaSBPs* in different organs of wheat showed that all *TaSBPs* expressed in root, stem and leaf. Of these, *TaSBP-A* had the highest expression level in three organs, particularly in the plant root (**Figure 1E**). Further RT-qPCR analysis displayed a similar expression pattern in different plant organs (**Figure 1F**). According to the above results, we chose *TaSBP-A* for further functional survey. *TaSBP-A* consisted of 1485 bp encoding 495 amino acid residues, and the deduced molecular mass was 54.1 kDa without considering protein modification.

To determine the subcellular location of the TaSBP-A, a *TaSBP-A*-*GFP* fusion vector (pTaSBP-A-GFP) driven by two CaMV35S promoter was constructed and transformed into wheat protoplasts. In cells only expressing GFP, the entire cytoplasm and nucleus were diffusely labeled by a laser scanning microscopy. In contrast, the GFP fluorescence of TaSBP-A-GFP was only visible at cytoplasm, which clearly indicates that the TaSBP-A was localized in the cytoplasm (**Figure 1G**).

### Cd stress response of TaSBP-A in wheat and overexpressed yeast cells

**Figure 2:**
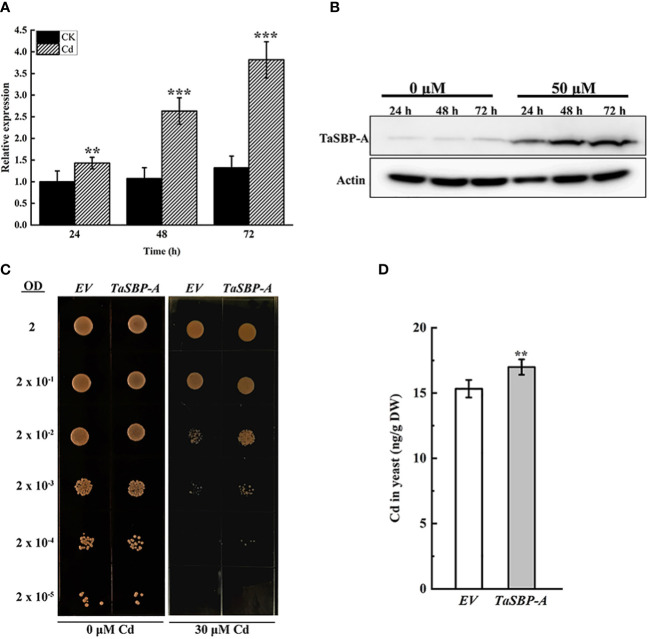
*TaSBP-A* responding to Cd-stress in Chinese Spring roots and its overexpression in *Saccharomyces cerevisiae* DEY1457. Wheat seedlings were treated with 50 μM CdCl_2_ for 24, 48 and 72 h during the three-leaf stage. **(A)** Transcription expression by RT-qPCR. **(B)** Protein accumulation by Western blot. **(C)** Serial dilutions of the *S. cerevisiae* strains expressing the pYES2 empty vector (EV); a pYES2 harbouring one of the *TaSBP-A* spotted on medium supplemented with 0 (control) or 30 μM CdCl_2_, as indicated at bottom of the panels. Each spot is comprised by 5 μl of a yeast culture diluted at the optical density at 600 nm (OD600 = 2.0 is the first spot). **(D)** Cd content of harvested cells measured by ICP-MS. A one-way ANOVA was used for the statistical analysis of the data. The asterisks represent the significant differences at different levels (**p<0.01; ***p<0.001).

The *TaSBP-A* expression in response to Cd stress in the seedling roots of Chinese Spring was detected by both RT-qPCR (**Figure 2A**) and Western blot (**Figure 2B**). The results showed that both transcription and translation expression levels of TaSBP-A were significantly induced by time increasing under Cd stress (**Figures 2A, B**). In particular, both transcription and translation of TaSBP-A arrived at the highest level at 72h compared with control (24h), suggesting that TaSBP-A can be induced and accumulated in roots by Cd stress and may have potential roles in Cd stress response.

Because yeast has no SBP homolog, we overexpressed the *TaSBP-A* in yeast to detect the Cd tolerance of yeast cells. As shown in **Figure 2C**, EV and *TaSBP-A* yeast cells had a practically equal growth rates under normal condition; however, the *TaSBP-A* cells showed a higher growth rate than did the *EV* cells under 30 μM Cd^2+^ treatment (**Figure 2C**). Subsequently, we respectively cultured *EV* and *TaSBP-A* cells in a liquid medium containing 25 μM CdCl_2_; here, the Cd content in yeast was detected by ICP-MS. Interestingly, the *TaSBP-A* overexpression in yeast could accumulate more Cd than EV significantly, as shown to be by 10.8% (**Figure 2D**). These results indicated that *TaSBP-A* can enhance the Cd tolerance of yeast cells by binding toxic free Cd ions.

### The constitutive heterologous expression of TaSBP-A alleviated the oxidative stress and photosynthesis impairment triggered by Cd treatment in Arabidopsis

To reveal *TaSBP-A* function in the response to Cd stress, we further overexpressed it in Arabidopsis. Three stable overexpressed transgenic lines (S2-9, S3-12 and S4-7) were generated using genome PCR and western blot detection (**Figures S2A-C**). The overexpression protein level of TaSBP-A is S4-7 < S2-9 <S3-12 (**Figure S2C**). The seedlings of the *TaSBP-A* transgenic lines and wild type (WT) were cultivated on 1/2 MS containing 0, 75, and 150 μM CdCl_2_ for 7 days. The primary root growth was significantly inhibited and substantial chlorosis of cotyledons occurred in the WT plants (**Figures S2D, E**). However, *TaSBP-A* overexpressed seedlings maintained a higher fresh weight, root length and survival rate (**Figures S2F-H**), showing that all of lines have a palpable Cd tolerance. The Cd accumulation measurement revealed that the overexpression of *TaSBP-A* lines could accumulate a greater amount of Cd, specifically 1.13-1.23 times as high as WT (**Figure S2I**).

**Figure 3:**
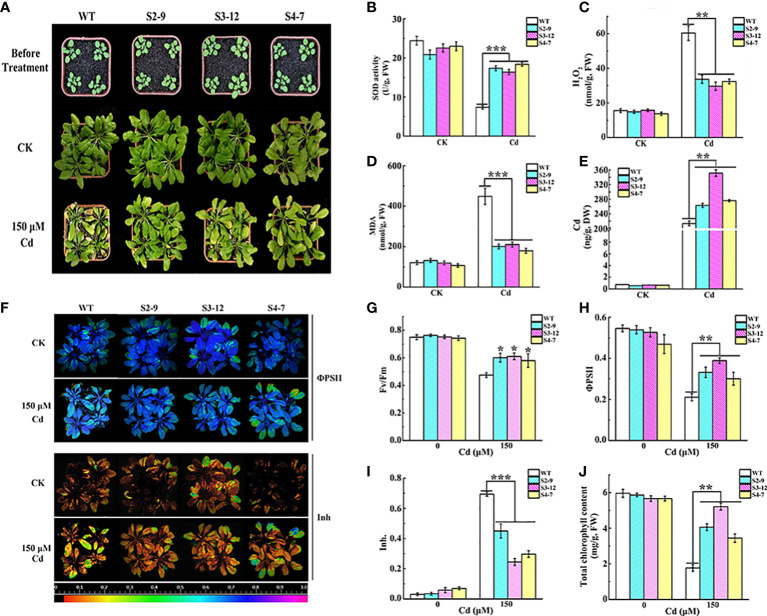
Overexpression of *TaSBP-A* alleviated the oxidative stress and photosynthesis impairment triggered by Cd treatment in Arabidopsis. **(A)** Phenotypic comparison of the wild type and transgenic Arabidopsis lines (S2-9, S3-12, and S4-7) under Cd stress. Three-week-old plants were watered with 150 μM CdCl_2_ once a week for four weeks. Control group (CK) was normally irrigated with water. **(B–D)** Changes of SOD activity, H_2_O_2_ and the MDA content of transgenic Arabidopsis and the wild type (WT). **(E)** The Cd content changes in the roots of transgenic and wild type lines. **(F)** Chlorophyll fluorescence changes of the efficiency of PSII in the light (ΦPSII), and the inhibition of the PSII quantum yield (Inh.) of WT and overexpressed Arabidopsis lines. **(G–I)** Changes of chlorophyll fluorescence parameters *F_v_/F_m_* **(G)**, ΦPSII **(H)**, and Inh **(I)** extracted from fluorescence images. **(J)** Chlorophyll content changes of WT and the transgenic line under Cd stress. The data are indicated as mean± SD from three biological replicates. All data were statistically analyzed using a one-way ANOVA. The asterisks represent significant differences at different levels (*p< 0.05; **p< 0.01; ***p< 0.001).

The Cd tolerance of *TaSBP-A* overexpressed lines grown in garden soil was further detected under 150 μM CdCl_2_ treatment. Both transgenic lines and WT had no clear differences in the growth under normal conditions. However, under Cd stress, the WT plants showed chlorosis leaves and a severe reduction of growth (**Figure 3A**). In contrast, the *TaSBP-A* transgenic plants displayed a clear resistance to Cd stress even though some chlorosis leaves still occurred. Physiological and biochemical parameter analyses showed that SOD activity in *TaSBP-A* overexpressed lines was 2.36 times and the content of H_2_O_2_ and MDA were only 50% and 39% as compared to WT plants (**Figures 3B-D**). These results demonstrated that *TaSBP-A* transgenic plants could effectively promote SOD activity that would be able to alleviate the oxidative stress triggered by heavy-metal-induced H_2_O_2_. Cd accumulation in the roots of ICP-MS showed that the *TaSBP-A* transgenic lines accumulated more Cd, 1.23-1.64 times higher than WT (**Figure 3E**).

We also detected the impairment of photosynthesis as a result of Cd stress in transgenic plants. The results showed that the chlorophyll fluorescence parameters had no clear differences between WT and *TaSBP-A* overexpressed lines under normal conditions (**Figure 3F**). However, the maximum quantum yield of PSII photochemistry in the dark‐adapted state (F_v_/F_m_) in the transgenic plant leaves under 150 μM Cd was higher than it was for WT (**Figure 3G**). In particular, the operating efficiency of PSII (ΦPSII) in three overexpression lines under Cd treatments was 22.25-28.80%, which was significantly higher than it was for WT (**Figures 3F, H**). Cd exposure significantly promoted the inhibition of PSII quantum yield (Inh) in the WT plant leaves, but it had no clear effects on transgenic plants (**Figures 3F, I**). Similarly, the chlorophyll content in the leaves had no significant differences between WT and the *TaSBP-A* transgenic lines after 4 weeks in normal growth conditions, while the *TaSBP-A* transgenic lines were significantly higher chlorophyll content under Cd stress: 1.95-2.95 times as high as WT (**Figure 3J**). These results indicate that the overexpression of *TaSBP-A* could significantly alleviated the photosynthesis impairment of plants under Cd stress treatment.

### Net fluxes and state of over-accumulated Cd^2+^ in transgenic Arabidopsis

**Figure 4:**
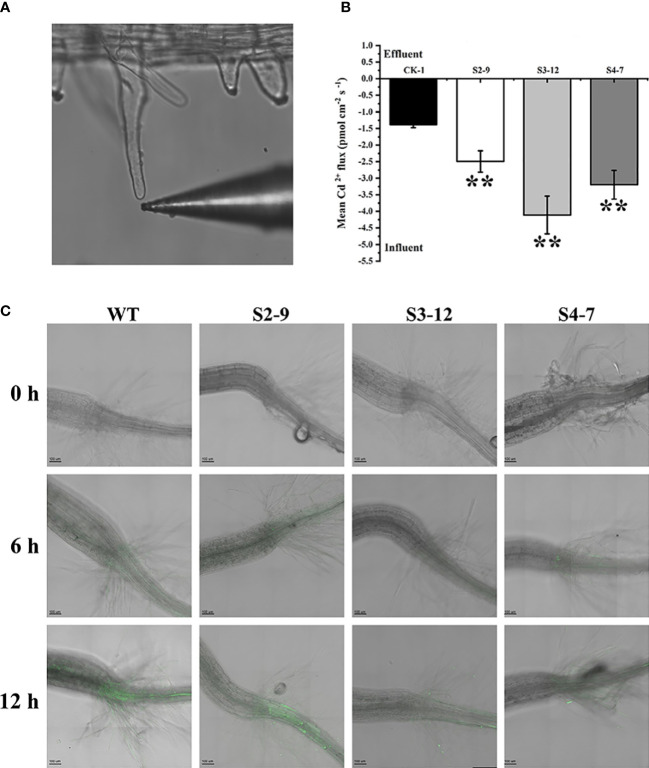
Cd^2+^ fluxes and free Cd^2+^ detection in *TaSBP-A* overexpressed Arabidopsis root hairs. **(A)** A representative root hair and the Cd^2+^-selective microelectrode used. **(B)** The mean Cd^2+^ fluxes in the root hair was measured for 30 min after exposure to 30 μM Cd^2+^ by using a noninvasive micro-test technique. The data are indicated as mean± SD from the three biological replicates. All data were statistically analyzed using a one-way ANOVA. The asterisks represent significant differences at different levels (*p< 0.05; **p< 0.01; ***p< 0.001). **(C)** Micrographs of seedling roots from WT and overexpressed lines exposed to 150 μM Cd for different treatment times. Plant roots were pre-treated with 150 μM CdCl_2_ for 0 h (control), 6 h and 12 h on a 1/2-MS plate and loaded with Leadamium™ Green AM dye for 60 min. All images were taken by a confocal laser scanning microscope (Leica TCS SP5, Germany). Green fluorescence indicates the binding of the dye to Cd.

As described above, *TaSBP-A* overexpression resulted in an increase of Cd accumulation in roots, thus we detected the net fluxes of Cd^2+^ in the root hairs of transgenic Arabidopsis under 30 μM Cd^2+^ using a noninvasive micro-test technique (**Figure 4A**). The results showed that the Cd^2+^ net fluxes in the root hairs of three transgenic lines S2-9, S3-12, and S4-7 were 2.49, 4.11 and 3.20 pmol cm^-2^·s^-1^ respectively, which is 1.8-2.9 times higher than that of WT (**Figure 4B**). This confirmed that the overexpression of *TaSBP-A* could enhance the Cd^2+^ accumulation in roots of plant.

Leadmium™ Green AM dye, a Cd probe, was used to detect the state (free or binding) of the accumulated Cd, particularly with regard to the distribution of free Cd^2+^ in plant roots. As shown in **Figure 4C**, a very low level of green fluorescence was found in the roots of transgenic lines. This indicates that this dye has a high specificity to detect Cd^2+^ and that it does not react with divalent ions such as the Ca^2+^ present in the control roots. In contrast, the green fluorescence signals in WT plants appeared clearly above the hypocotyl after a 6 h pretreatment of 150 μM Cd^2+^; they were more obviously enhanced after a 12 h pretreatment of 150 μM Cd in WT plants. However, the slight green fluorescence signals in the overexpression lines were only present below hypocotyl. These results indicated that overexpressed *TaSBP-A* played an important role in reducing free Cd^2+^ and that the over-accumulated Cd in the *TaSBP-A* overexpression lines was present largely in binding state.

### Overexpressing TaSBP-A enhanced Cd tolerance by inhibiting the transfer of Cd from root to leaf in wheat seedlings

**Figure 5:**
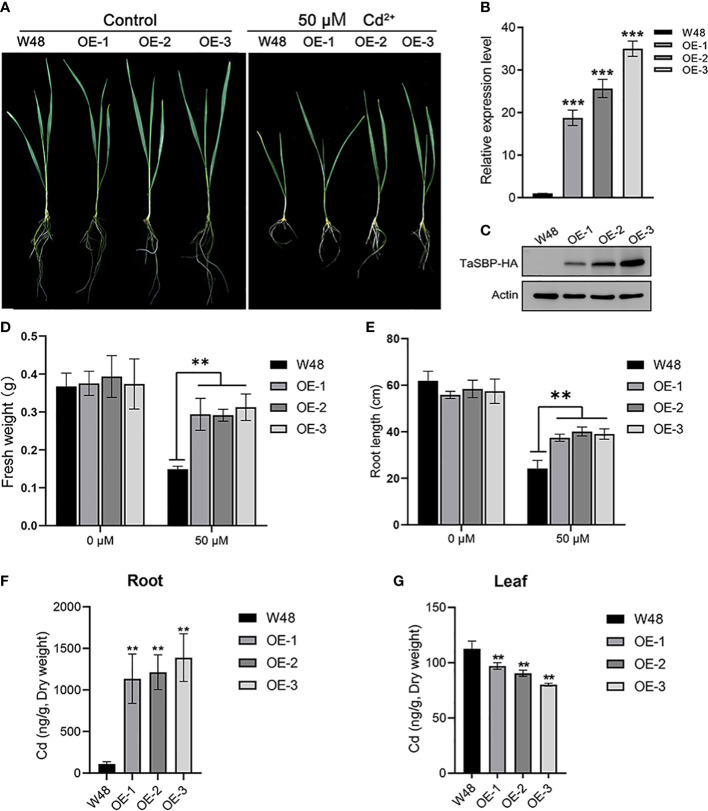
Overexpression of *TaSBP-A* enhanced wheat tolerance to Cd stress. **(A)** Seedlings of W48 and three *TaSBP-A* overexpressed lines (OE-1, OE-2 and OE-3) under 50 μM Cd^2+^ treatment for two weeks. **(B)** The expression levels of *TaSBP-A* relative to the internal control *UBI* gene in W48, OE-1, OE-2 and OE-3 determined by RT-qPCR. **(C)** The protein level of TaSBP-A-HA in leaf of W48 and *TaSBP-A* overexpressed wheat seedlings were validated using Western blot. **(D)** The fresh weight of plants under Cd stress. **(E)** The root length of plants under Cd stress. **(F)** The content of Cd in plant roots under 50 μM Cd^2+^ treatment for two weeks. **(G)** The content of Cd in plant leaves under 50 μM Cd^2+^ treatment for two weeks. The data are shown in mean values ± Sd. One-way ANOVA was used for statistical analysis of all data. The asterisks represent significant differences at different levels (**p< 0.01, ***p< 0.001).

Three stable *TaSBP-A* overexpressed wheat lines, OVEREXPRESSION-1 (OE-1), OE-2 and OE-3, were obtained at T3 generation. The RT-qPCR and Western blot analysis showed both the transcription and translation expression levels of TaSBP-A were significantly higher in *TaSBP-A* overexpressed wheat seedlings than those in W48 seedlings (**Figures 5B, C**). The growth of W48 and *TaSBP-A* overexpressed wheat seedlings exposed to 0 and 50 μM CdCl_2_ was compared after two weeks. Under normal condition, W48 and *TaSBP-A* overexpressed wheat seedlings showed a similar morphological characteristics (**Figure 5A**), and significant differences were not found in the fresh weight and root length between W48 and *TaSBP-A* overexpressed lines (**Figure 5D, E**). When subjected to 50 μM CdCl_2_ treatment, a repressed growth was observed in both W48 and *TaSBP-A* overexpressed wheat seedlings, but W48 seedlings were more sensitive to Cd treatment (**Figure 5A**). The statistical analysis showed that both the fresh weight and root length of *TaSBP-A* overexpressed seedlings were higher than those in W48 seedlings (**Figure 5D, E**), indicating that *TaSBP-A* overexpressed wheat seedlings have a higher tolerance to Cd stress. In addition, the measurement of Cd content showed that a significantly higher amount of Cd was accumulated in the roots of *TaSBP-A* overexpressed wheat seedlings compared with W48 (**Figure 5F**). However, a lower amount of Cd was accumulated in the leaf of *TaSBP-A* overexpressed wheat seedlings (**Figure 5G**). These results indicated that overexpressed *TaSBP-A* could improve Cd tolerance of wheat seedlings by inhibiting the transfer of Cd from the root to leaf in wheat seedlings.

### Determination of Cd-binding site in TaSBP-A

**Figure 6:**
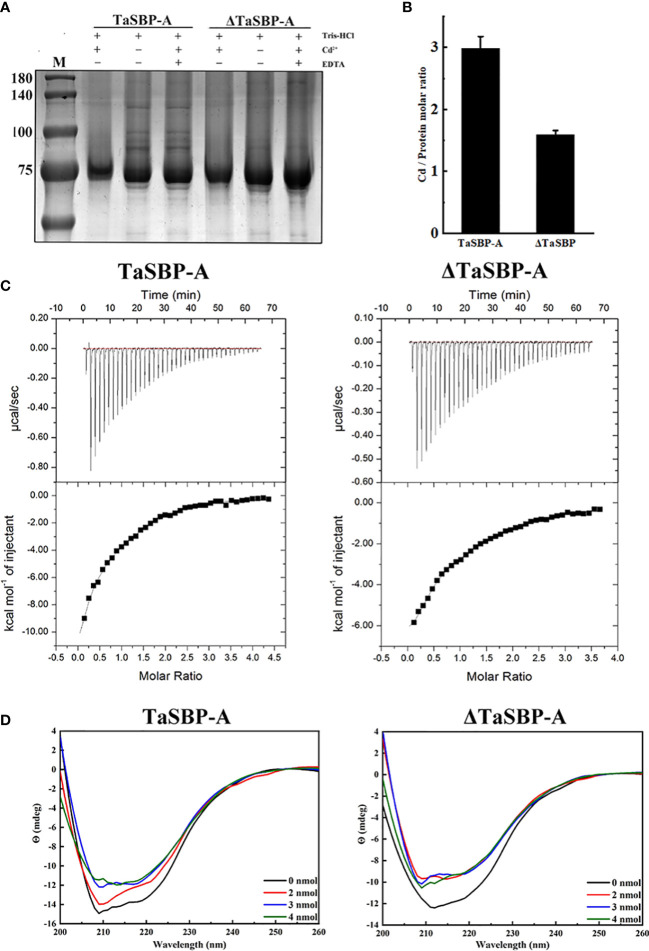
Determination of the Cd-binding site in TaSBP-A by *in vitro* Cd^2+^ binding assay. **(A)** Cd^2+^-binding shift performed with 100 μM recombinant proteins and 0.5 mM Cd^2+^ *in vitro*. Tris-HCl was used as a reaction buffer and EDTA was used as a competitive inhibitor to protein. **(B)** The Cd/Protein Ratio determined by ICP-MS. **(C)** Isothermal titration calorimetry experiments of the recombinant TaSBP-A (left) and ΔTaSBP-A (right) binding to Cd. The top panel dispalys the titration of Cd^2+^ at 0.5 mM into TaSBP-A (ΔTaSBP-A) at 50 μM placed at the sample cell; the bottom panel indicates the ligand concentration dependence of the heat released upon binding after normalization. The data were fitted with the one-site binding model. The data are means ± SD from the three biological replicates. **(D)** CD spectra of the recombinant TaSBP-A (left) and ΔTaSBP-A (right) in the presence of a stepped Cd concentration (0-4 nmol Cd) at 25 °C. The protein content remained 1 nmol/250 μL during the whole experiment. The X and Y axes represent the wavelength (nm) and Y (mdeg), respectively.

The above results indicated that TaSBP-A can enhance Cd tolerance of plants *via* reducing free Cd^2+^ and inhibiting the transfer of Cd from root to leaf. Thus, it is crucial to determine the Cd binding site in TaSBP-A in order to dissect the molecular mechanism of plant Cd-tolerance. The putative heavy metal binding motif CXXC (TaSBP-A) was mutated to GXXG (ΔTaSBP-A) and then the TaSBP-A and ΔTaSBP-A were respectively expressed in *E. coli* as a fusion protein with GST (**Figure S3A**). Subcellular localization indicated that the mutant of CXXC had no influence on the location of TaSBP-A (**Figure S3B**). Thus, we further conducted an *in vitro* Cd^2+^ binding assay according to the principle that ethylenediaminetetraacetic acid (EDTA) can chelate with Cd^2+^ to form Cd (II)-EDTA (Yang and Davis, 1999). As shown in **Figure 6A**, both recombinant TaSBP-A and ΔTaSBP-A had shifts in SDS-PAGE after incubation with 500 μM CdCl_2_; in contrast, the shift disappeared with EDTA and the Cd coexistence in incubation buffer. However, after binding Cd^2+^, it was hard to recognize the shift between TaSBP-A and ΔTaSBP-A. ICP-MS combined with protein quantification was further used to identify the stoichiometry of the interaction between Cd and protein, and the results showed that the stoichiometry of the TaSBP-A:ΔTaSBP-A binding to Cd was about 3: 2 (2.98: 1.59) (**Figure 6B**). This indicates that the ability of TaSBP-A decreased by 47.3% after the CXXC motif mutation. This result further witnessed the interaction between TaSBP-A with Cd, where the CXXC motif served as the heavy metal binding site in the TaSBP-A.

### Thermodynamic parameter analysis of the Cd^2+^ interactions with TaSBP-A and the impact of Cd^2+^-binding on TaSBP-A’s secondary structure

The interaction of Cd^2+^ with TaSBP-A and ΔTaSBP-A was detected by ITC. The ITC curves and thermodynamic parameters during the interaction of Cd^2+^ and TaSBP-A/ΔTaSBP-A are shown in **Figure 6C** and **Table 1**. For each Cd^2+^ injection, a release of heat was observed (**Figure 6C**, upper panel); this indicates that a clear binding event between Cd^2+^ and TaSBP-A/ΔTaSBP-A occurred. A one-site binding model was used to fit TaSBP-A/ΔTaSBP-A isotherms (**Figure 6C**, lower panel). This can determine an apparent binding affinity constant (K) in the low micromolar range (50 μM). Unexpectedly, the binding enthalpy value of the Cd^2+^ and TaSBP-A interaction was -17.92 kcal/mol, which was significantly higher than that of the Cd^2+^ and ΔTaSBP-A interaction (-13.30 kcal/mol) (**Table 1**). Therefore, we deduced that the two different strong negative enthalpy values (ΔH) for Cd^2+^ most likely correspond to a combination of a binding event with a specific covalent reaction between TaSBP-A/ΔTaSBP-A and Cd^2+^. However, less heat was released in the interaction of ΔTaSBP-A with Cd^2+^, which suggests that the Cd binding amount of TaSBP-A was palpably diminished after the mutation of the CXXC motif. The CXXC motif also witnessed entropy difference values (ΔS), a measurement for disorder and order in atomic and molecular assemblies (Landsberg, 1984). According to ΔS_TaSBP-A_ < ΔS_ΔTaSBP-A_, TaSBP-A was able to bind more Cd^2+^ than that of ΔTaSBP-A, leading to a dramatic decrease in ΔS. Furthermore, the calorimetric signals needed abnormally large amounts of time to recover to their baseline values after the CXXC motif mutation (**Figure 6C**) suggest that a covalent reaction combined with the binding of the ion was significant restrained due to the CXXC motif mutation in TaSBP-A. According to the K value, K_TaSBP-A_ was 1.6 times as high as K_ΔTaSBP-A_ (**Table 1**), which is consistent with the results of the stoichiometry TaSBP-A/ΔTaSBP-A (**Figure 6B**). This indicates that the ability of TaSBP-A to bind to Cd was heavily suppressed after the loss of CXXC motif, which can reduce the affinity of other binding sites in TaSBP-A to Cd^2+^.

**Table 1:** Thermodynamic parameters calculated from micro-calorimetric experiments for the interaction between Cd^2+^ and TaSBP-A/ΔTaSBP-A at 25°C (298.15 K) in 10 mM HEPES, pH 7.4, 150 mM NaCl.

Proteins	K (M^-1^)	ΔH (kcal/mol)		ΔS (cal/mol/K)	ΔG (kcal/mol)
TaSBP-A	(33.2 ± 3.49) x 10^3^	-17.92 ± 1.93		-39.4	-6.17 ± 1.93
ΔTaSBP-A	(20.7 ± 1.51) x 10^3^	-13.30 ± 1.11		-24.9	-5.88 ± 1.12

CD analysis was used to further determine whether Cd-binding caused changes of the protein’s secondary structure. The CD spectrum recorded the far-UV with TaSBP-A, ΔTaSBP-A, Cd-bound TaSBP-A and Cd-bound ΔTaSBP-A (**Figure 6D**) to estimate the second structure of proteins and Cd-proteins complexes. According to **Table S1**, the mutation of the CXXC motif had no effects on the *β*-sheet (about 45%) and turn (about 7.4%) content, but it resulted in an increase in random coil and a decrease in *α*-helix. With the presence of Cd^2+^, the spectrum of TaSBP-A had more significant changes than did ΔTaSBP-A, indicating that the Cd-binding to TaSBP-A and the complex formation led to clear second structure alterations (**Figure 6D**). According to **Table S1**, the changes of TaSBP-A were obviously observed in the *α*-helix, *β*-sheet, turn and random coil content after binding to Cd^2+^; in contrast, ΔTaSBP-A had only changes in turn content. These results showed that the significant changes of TaSBP-A’s second structure occurred upon Cd^2+^ binding. Moreover, the CXXC motif was a crucial Cd^2+^-binding site which could cause significant alterations of the protein’s second structure as a result of interaction between TaSBP-A and Cd^2+^.

### *In vivo* validation of Cd-binding CXXC motif in wheat protoplasts

We transformed *TaSBP-A* and *ΔTaSBP-A* to wheat protoplasts for further Cd-binding CXXC motif and function verification of TaSBP (**Figure S4**). Empty vector 16318hGFP and recombinant plasmids containing *TaSBP-A* and *ΔTaSBP-A* were transformed into wheat leaves protoplasts, and then they were cultured with protoplasts culture medium with 50 μM Cd^2+^ (**Figure S4A**). The viable protoplasts (yellow light overlapped by chloroplast autofluorescence and GFP green fluorescence) that occurred after transformation were counted (**Figure S4B**). The transgenic efficiency of the empty vector was 27%, and it remained stable during the whole experiment under normal condition (0 μM Cd^2+^). In turn, *TaSBP-A/ΔTaSBP-A* had a transform efficient of approximately 20%, serving as the basis to explore the relative rate of the protoplasts’ viability (RRPV) under Cd treatment (**Figure S4A**). Compared to TaSBP-A (73%) and *ΔTaSBP-A* (56%), the number of viable protoplasts dramatically dropped after 12 h of Cd treatment in the empty vector, during which RRPV declined to 25% (**Figure S4B**). The dynamic RRPV was plotted from 0-12 h under cadmium treatment and it was also linearized (**Figure 4B**, **Table S2**). The results showed an order of Slope_EV_ < Slope_ΔTaSBP-A_ < Slope_TaSBP-A_ (-0.06306< -0.04225< -0.02226) (**Table S2**), which indicates that the death rate of protoplasts was EV> ΔTaSBP-A> TaSBP-A. Compared to *TaSBP-A*, the dynamic RRPV of *ΔTaSBP-A* transgenic cells during 0-4 h had no significant difference. However, after 4 h under Cd stress, the reduction of *ΔTaSBP-A* RRPV was more palpable than that of *TaSBP-A* (**Figure S4**). These results revealed that the CXXC motif played a key role in TaSBP tolerance to Cd stress, whose mutation can heavily inhibit the ability of TaSBP to interact with Cd^2+^.

## Discussion

Abiotic stresses are the major adverse factors affecting crop yield. Thus, it is highly important for crop genetic improvement to discover potential stress-resistant genes and to explore the molecular mechanisms of plant adverse response. In particular, Cd severely affects the plant metabolic and physiological processes through elevating ROS (Ranieri et al., 2005) and through cell ultrastructural damages (Chen et al., 2018; Cheng et al., 2018). It can also enter chloroplasts and disturb chloroplast function by inhibiting the enzymatic activities in the chlorophyll biosynthesis and Calvin cycle, which leads to a decrease of chlorophyll content and photosynthesis (Ying et al., 2010). At the same time, excess Cd can cause overproduction of MDA content in wheat shoots and roots (Chen et al., 2010).

Plant SBPs can be induced by various stressors such as Cd, Se, Cu, Zn, and H_2_O_2_ (Dutilleul et al., 2008; Hugouvieux et al., 2009). We found that *TaSBP* genes expressed in different wheat organs; in particular, *TaSBP-A* had the highest expression level in the roots (**Figures 1E, F**). Similar to *AtSBP1* in Arabidopsis (Sarry et al., 2006; Dutilleul et al., 2008), *TaSBP-A*, as a highly hydrophilic cytosolic protein, was highly induced by Cd stress (**Figure 2A, B**). Its overexpression of yeast (**Figures 2C, D**), Arabidopsis (**Figure S2**, **Figures 3**, **4**) and wheat (**Figure 5**) conferred Cd-tolerance through reducing free Cd^2+^ and inhibiting the transfer of Cd from root to leaf in plants. In addition, its overexpression contributed to photosynthesis impairment alleviation and ROS scavenging in Arabidopsis in our study. AtSBP1 protein could interact with AtGRXS14, which functions in redox state regulation in the chloroplasts (Valassakis et al., 2019). Taken together, the overexpression of TaSBP-A might enhance Cd tolerance in plant by two ways: one is regulation of redox state in chloroplasts by interaction with GRXS; the other is specific Cd-binding site present in TaSBP-A that directly interact with Cd to form a protein complex and, subsequently, to alleviate Cd toxicity.

The Cd toxicities are mainly brought up by free Cd^2+^, which has a high ability to substitute other metals to serve as crucial active centers such as Cu in SOD and Mg in chlorophyll (Choppala et al., 2014). The loading of free Cd^2+^ into the root xylem can be mediated by heavy metal P_1B_-ATPase, such as orthologues of HMA2 and HMA4 (Mendoza-Cózatl et al., 2011; Ismael et al., 2019). To date, the binding ability of SBPs to kinds of heavy metal such as Cd^2+^, Zn^2+^ and Ni^2+^ has been reported by multiple researchers (Dutilleul et al., 2008; Schild et al., 2014). In this study, our results confirmed that *TaSBP-A* overexpression significantly reduced free Cd^2+^ content in plant roots (**Figure 4C**) as well as reduction of Cd content in wheat leaves (**Figure 5G**), which indicated that its overexpression might impede Cd long-distance translocation by chelation of free Cd^2+^ and lead to lower Cd content in overexpression wheat leaves than WT. Contaminated wheat and its products are some of the essential food contributors to dietary Cd intake by people (Abbas et al., 2017). Thus, it suggested that TaSBP may have potential to reduce Cd accumulation in grains.

In this study, we found the major existence of over-accumulated Cd occurred in the Cd-complexes (**Figure 4**), suggesting its strong binding ability to Cd. As a cytosolic protein, SBPs have a putative heavy metal binding motif CXXC that is highly conservative among different plant species (**Figure S1**). This motif contained two free accessible Cys residues on the random coils (**Figure 1D**), which may facilitate Cd-binding and protein complex formation. Many proteins containing the CXXC motif have been found to involve in metal ion metabolism and detoxification; this includes Cd19, MerP and ATX1 (Lin and Culotta, 1995; Powlowski and Sahlman, 1999; Suzuki et al., 2002). In this study, we provided sufficient evidence to confirm that the CXXC motif in TaSBP-A serves as a major Cd-binding site that can interact with Cd and form a metal complex to reduce the free Cd^2+^ content in root and decreased the amount of Cd^2+^ transferred from root to leaf in plants, and therefore alleviate the oxidative stress and photosynthesis impairment triggered by Cd stress.

We noticed that the mutation of CXXC to GXXG (ΔTaSBP-A) still had Cd-binding ability that caused some changes of the thermodynamic properties and the secondary structure of the recombinant proteins (**Figures 6C, D**; **Table S1**). This suggests that, in addition to the main CXXC binding site in TaSBP-A, other metal binding sites are still likely to be present. The side-chain carboxylate, sulfur and imidazole groups generally dominate metal coordination in proteins such as histidine, aspartic acid, glutamic acid and cysteine (Tainer et al., 1992). In particular, cysteine residues such as Cys^21^Cys^22^ for Se-binding in AtSBP1 were found to have binding ability (Schild et al., 2014). In addition, TaSBP-A had two more cysteine residues (Cys^51^ and Cys^289^) than did AtSBPs. Thus, it can be excluded from the Cd-binding candidate sites with high probability due to its non-conservative characteristics (**Figure S5**). The *β*-sheet forms the framework for proteins and the loop between two *β*-sheets has high flexibility (Chothia et al., 1998). According to the predicted 3-D structure of TaSBP-A (**Figure 1D**), seven β-sheets provide the structural framework of TaSBP-A; in turn, this may cause more rigid structure for the residues located on *β*-sheet. Thus, the Cys^161^ and Cys^171^ located on β-sheet may be less flexible for Cd^2+^-binding. The left cysteine residues (Cys^23^, Cys^24^ and Cys^488^) may serve as the candidate Cd-binding sites. In addition, three His-rich motifs (two HxD and one HxxH) are highly conserved in both TaSBPs and AtSBPs (**Figure S5**); as such, they may also serve as potential heavy metal binding sites (Flemetakis et al., 2002; She et al., 2003; Agalou et al., 2006). Further studies, however, are still needed to determine if this is the case.

In comparison with several micro-nutrients such as Zn, Mn and Ni, Cd translocation moves slowly from the root system to the shoot (Choppala et al., 2014). Almost 50% of the absorbed Cd is retained in the plant roots (Obata and Umebayashi, 1993). Harmful excess metal ions may enter cells *via* the cation transporters of the root tissues (Thomine et al., 2000). In order to deal with the damage inflicted by these absorbed metals, plants can produce metallochaperones to maintain appropriate levels of the metal concentration by binding and releasing (Suzuki et al., 2002). Subsequently, the bound metals may be transferred to metal trapping compounds such as phytochelatins, finally detoxifying them in vacuoles (Sanità Di Toppi and Gabbrielli, 1999; Cobbett and Goldsbrough, 2002; Wang et al., 2015). In this study, we found that the overexpression of *TaSBP-A* can cause higher absorption rates of Cd^2+^ as well as Cd^2+^-locking under the hypocotyl of plant roots. Thus, TaSBP-A may serve as a cytoplasmic heavy metal transporter (metallochaperones) to help accelerate metal ion transport into the vacuole since the SBP56 family protein has a transport function that is involved in intra-Golgi protein transport (Porat et al., 2000).

**Figure 7:**
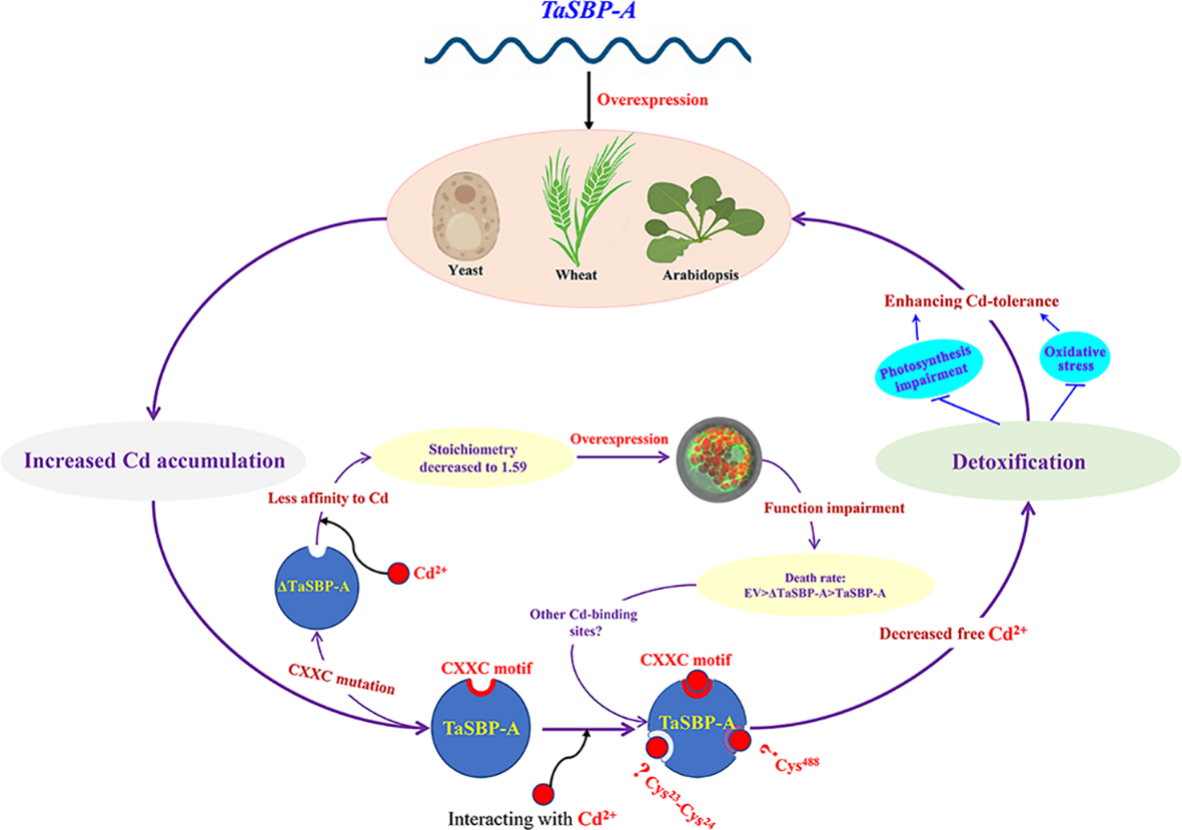
Schematic representation of TaSBP-A involved in Cd^2+^-binding and detoxification in plants. The overexpression of *TaSBP-A* confers Cd-tolerance in yeast, Arabidopsis and wheat through the detoxification of free Cd^2+^. The interaction between the CXXC motif and Cd enhances the sequestration of free Cd^2+^, inhibits the Cd transfer from root to leaf and reduces the content of Cd^2+^ in plant leaf, ultimately conferring plant Cd-tolerance *via* alleviating the oxidative stress and photosynthesis impairment triggered by Cd stress.

In conclusion, as a cytoplasmic protein and typical SBP56 family member, TaSBP-A highly expressed in plant roots and it was highly induced by Cd stress. The overexpression of *TaSBP-A* conferred Cd-tolerance in yeast, Arabidopsis and wheat through the detoxification of free Cd^2+^. The CXXC motif in TaSBP-A was confirmed as a major Cd-binding site *via* an *in vitro* Cd^2+^ binding assay in combination with a thermodynamics survey and a secondary structure analysis. The interaction between the CXXC motif and Cd as well as the metal protein complex formation enhanced detoxification of free Cd^2+^, inhibited the Cd transfer from root to leaf and reduced the content of Cd^2+^ in plant leaf, ultimately conferring plant Cd-tolerance *via* alleviating the oxidative stress and photosynthesis impairment triggered by Cd stress (**Figure 7**).

## Data availability statement

The datasets presented in this study can be found in online repositories. The names of the repository/repositories and accession number(s) can be found in the article/**Supplementary Material**.

## Author contributions

FL, DZ and HS: Investigation, Writing-Original draft preparation. WD and JL: Investigation. YY: Conceptualization, Supervision, Writing- Reviewing and Editing. All authors contributed to the article and approved the submitted version.
