# Supplementary material for: Wheat Selenium-binding protein TaSBP-A enhances cadmium tolerance by decreasing free Cd2+ and alleviating the oxidative damage and photosynthesis impairment
Source: Front Plant Sci. 2023 Feb 7;14:1103241. doi: 10.3389/fpls.2023.1103241 (PMC9941557; doi:10.3389/fpls.2023.1103241)
Supplement: Supplementary file 1 [file DataSheet_1.docx]

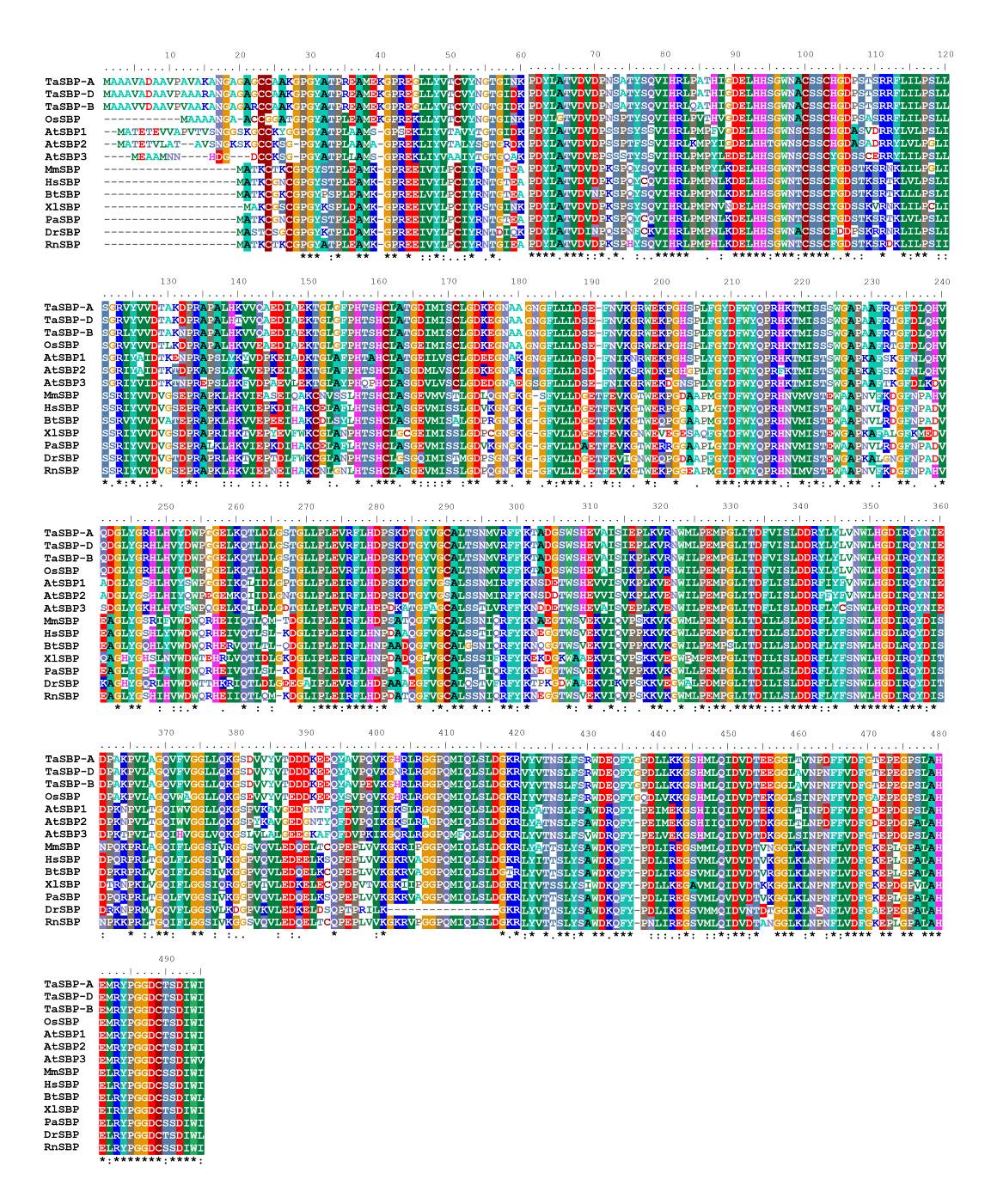


**Figure S1. Multiple sequences alignment of the deduced amino acid of the SBP proteins of various species.** The alignment was performed using the ClustalW method. The asterisks indicate identical residues while gaps in the alignment are represented by dashes. The similarity in wheat of TaSBP-A with TaSBP-B and TaSBP-D is 98.59% and 97.37%, respectively. The SBP proteins from different organisms were highly conserved. Os (*Oryza sativa subsp*, Q8RZW7), At (*Arabidopsis thaliana*, O23264, Q93WN0, Q9LK38), Mm (*Mus musculus*, P17563), Hs (*Homo sapiens*, Q13228), Bt (*Bos Taurus*, Q2KJ32), Xl (*Xenopus laevis*, Q52KZ7), Pa (*Pongo abelii*, Q5RF48), Dr (*Danio rerio*, Q6PHD9) and Rn (*Rattus norvegicus*, Q8VIF7).


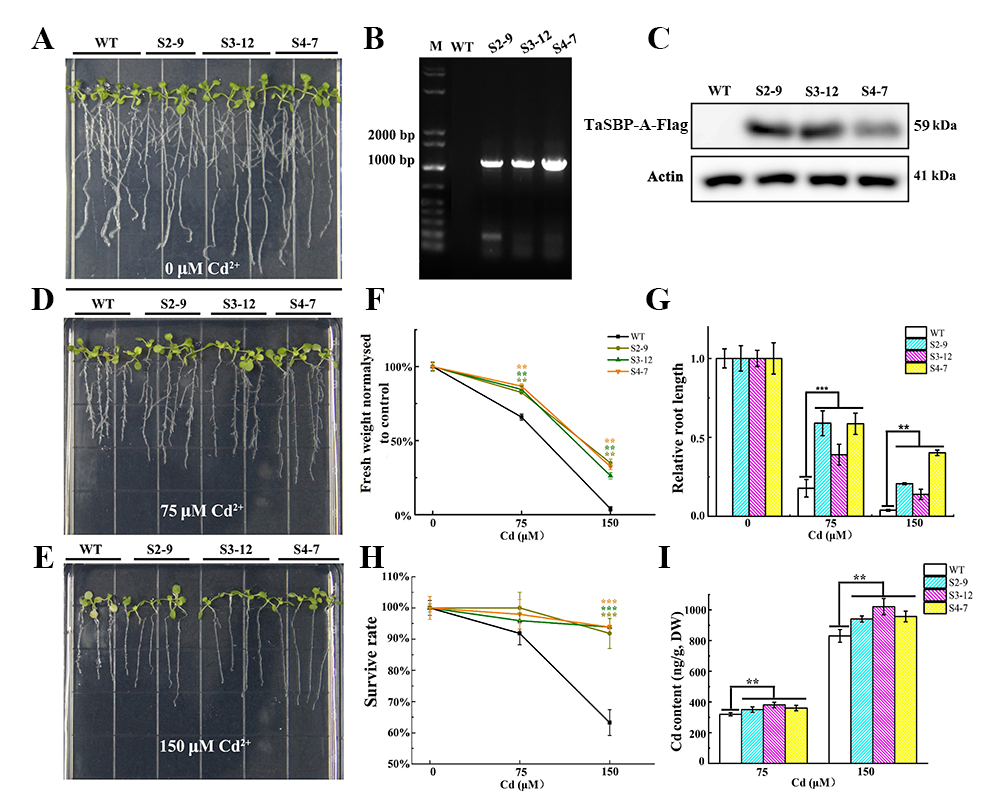


**Figure S2. Estimation of the Cd-tolerance of *TaSBP-A* overexpressed Arabidopsis seedlings.** **(A)** Phenotypic observation of wild type and *TaSBP-A* transgenic seedlings (S2-9, S3-12 and S4-7) under normal condition. **(B)** A pair of chimeric primers were designed to verify the integration of *TaSBP-A* into Arabidopsis’s genome. PCR was performed with the template strand of Arabidopsis’s genome. **(C)** The protein level of TaSBP-A-Flag in the transgenic lines was validated using Western blot. **(D and E)** Phenotypic observation of wild type and *TaSBP-A* transgenic seedlings (S2-9, S3-12 and S4-7) under Cd stress. Five-day-old seedlings were treated with different CdCl_2_ concentrations (75 and 150 μM Cd^2+^) for 7 days. **(F)** The relative fresh weight of plants under Cd stress. **(G)** The relative root length of plants under Cd stress. **(H)** The survival rate of *TaSBP-A* transgenic and wild type seeds on 1/2 MS with different CdCl_2_ (0-150 μM). **(I)** Total Cd content of 5-day-old seedlings of the transgenic and wild type lines with 75 and 150 μM Cd treatments for 7 d, as determined by ICP-MS. The data are shown in mean values ±SD. One-way ANOVA was used for statistical analysis of all data. The asterisks represent significant differences at different levels (**p*<0.05; ***p*<0.01; ****p*<0.001).


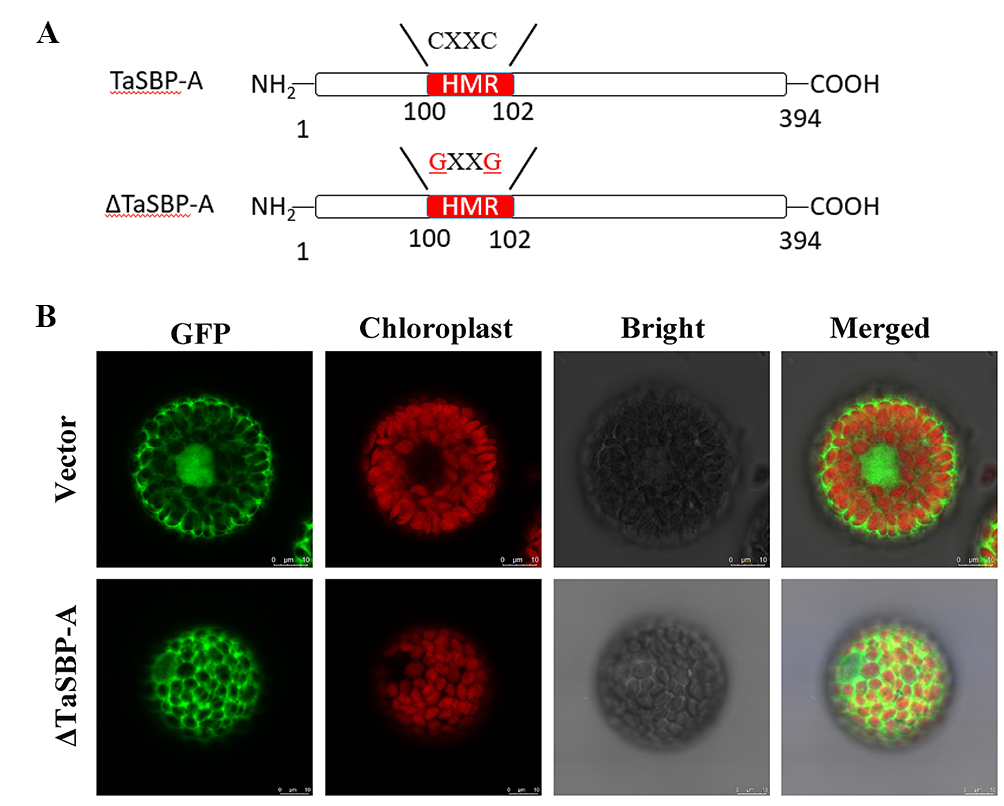


**Figure S3. Illustration of the recombinant TaSBP-A and ΔTaSBP-A proteins and the subcellular localization of ΔTaSBP-A**. **(A)** Schematic diagrams of the recombinant proteins containing the metal binding regions of TaSBP-A and mutant (ΔTaSBP-A) expressed in *E. coli*. The mutant (ΔTaSBP-A) contains displaced Cys with Gly in the metal binding regions. **(B)** Subcellular distribution of the 35S-ΔTaSBP-GFP fusion proteins in *Chinese Spring* leaf protoplasts, observed using a confocal laser scanning microscope. The red is chloroplast fluorescence and the green is GFP fluorescence.


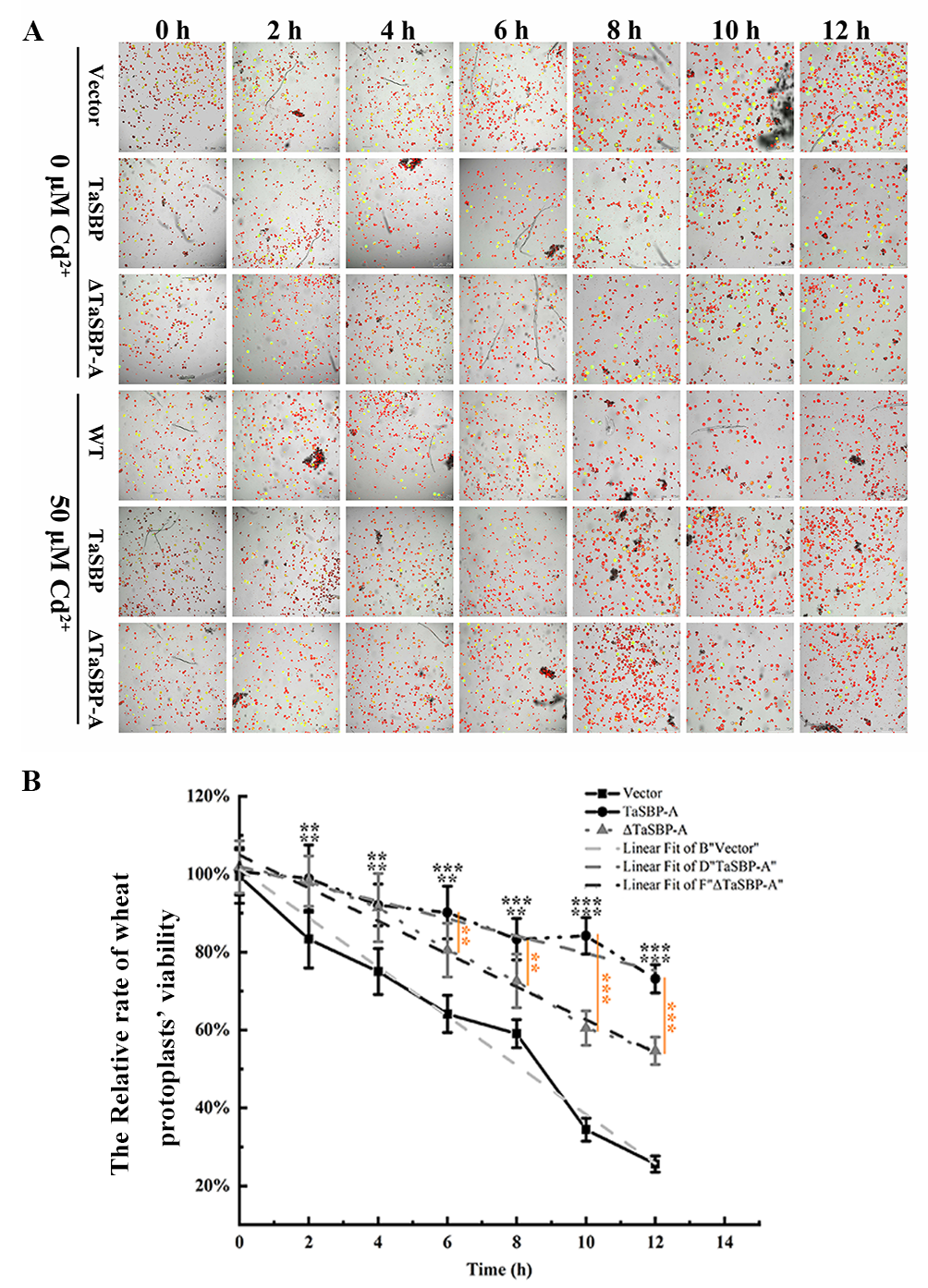


**Figure S4. The viability comparison of *TaSBP-A* and *ΔTaSBP-A* overexpressed wheat protoplasts under Cd treatment.** **(A)** The viability of the wheat protoplasts at 0-12 h after treatment with 50 μM Cd. The 16318hGFP empty vector, 16318-*TaSBP-A* and 16318-*ΔTaSBP-A* were transformed into wheat protoplasts and then treated with 50 μM CdCl_2_. All images were taken using a Leica TCS SP5. The viable protoplasts appear yellow due to the overlay of both red (chloroplast autofluorescence) and green (GFP); the non-viable protoplasts appear red from the chloroplast autofluorescence. **(B)** The relative rate of wheat protoplasts’ viability accompanied with yellow at 0-12 h under 50 μM CdCl_2_ treatment. The CK group was cultured without Cd. One-way ANOVA was used for statistical analysis of all data obtained. The asterisks represent significant differences at different levels (**p*< 0.05; ***p*< 0.01; ****p*< 0.001).


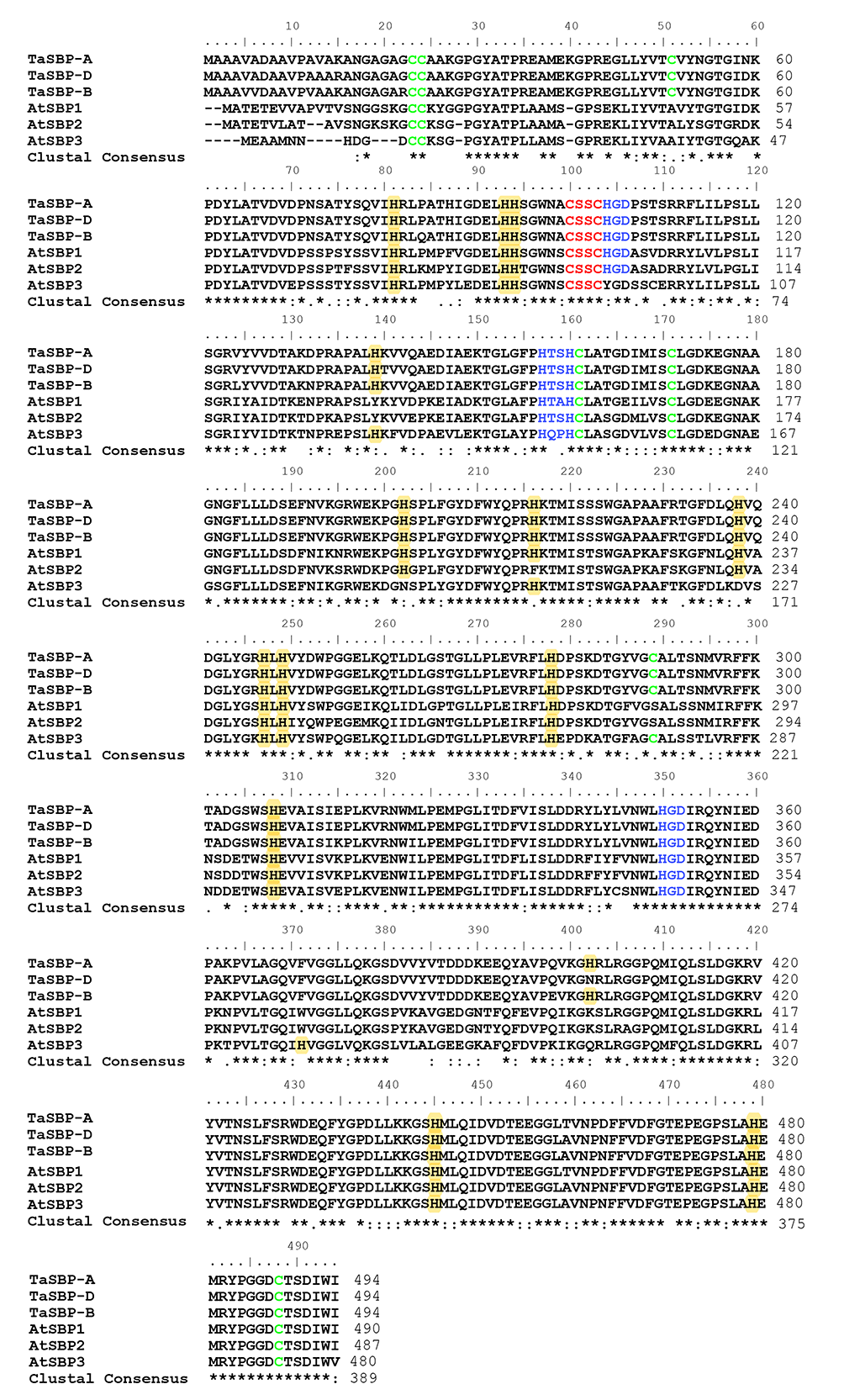


**Figure S5. Comparison of the potential amino acid sequences of SBP proteins that may serve as heavy metal binding sites.** The alignment was performed using the ClustalW method. The asterisks indicate identical residues while gaps in the alignment are represented by dashes. The witnessed CSSC sequence motif is highlighted in red and the putative metal-binding sites HxD and HxxH are highlighted in blue. Additional conserved Cys residues are highlighted in green while His residues are highlighted in yellow.

**Table S1.** Effects of Cd-binding on the secondary structure of TaSBP-A and ΔTaSBP-A proteins.

| Molar ratio (Protein/CdCl_2_) | | Second structural contents in TaSBP-A | | | | | |
| --- | --- | --- | --- | --- | --- | --- | --- |
|  |  | α-helix | β-sheet | Turn | Random | |  |
| TaSBP-A | 1:00 | 28.2 ± 0.7 | 45.0 ± 0.3 | 7.4 ± 0.4 | | 19.4 ± 1.4 | |
|  | 1:02 | 22.2 ± 0.8 | 37.3 ± 4.0 | 10.7 ± 2.5 | | 29.9 ± 0.8 | |
|  | 1:03 | 20.8 ± 2.4 | 46.4 ± 3.4 | 7.2 ± 3.1 | | 25.6 ± 2.1 | |
|  | 1:04 | 19.5 ± 3.7 | 45.3 ± 6.1 | 7.8 ± 4.7 | | 27.5 ± 2.3 | |
| ΔTaSBP-A | 1:00 | 18.6 ± 1.4 | 45.0 ± 0.4 | 7.8 ± 0.7 | | 28.6 ± 2.1 | |
|  | 1:02 | 19.2 ± 1.1 | 43.1 ± 5.0 | 13.5 ± 2.6 | | 24.4 ± 1.3 | |
|  | 1:03 | 18.7 ± 1.7 | 40.6 ± 2.4 | 14.2 ± 0.4 | | 26.7 ± 3.7 | |
|  | 1:04 | 17.5 ± 0.3 | 38.6 ± 1.8 | 14.5 ± 1.1 | | 29.5 ± 0.2 | |

**Table S2.** Linearization of data in Figure 8b.

|  | Intercept | | Slope | | | Statistics |  |
| --- | --- | --- | --- | --- | --- | --- | --- |
|  | Value | Standard Error | Value | Standard Error | Adj. R-Square | | |
| Vector | 1.01411 | 4.91% | -0.06306 | 0.005 | | 0.96338 |  |
| TaSBP-A | 1.01977 | 2.23% | -0.02226 | 0.00258 | | 0.92471 |  |
| ΔTaSBP-A | 1.04917 | 1.74% | -0.04225 | 0.00192 | | 0.98777 |  |

**Table S3.** The information of primers for PCR, RT-qPCR and site-directed mutation.

| **Primer** | **Sequence 5'-3'*** | **Restriction Enzyme cutting site** |
| --- | --- | --- |
| *TaSBP*-16318-F | tatctctagaggatccATGGCCGCAGCGGTGGCTGA | *BamH*Ⅰ |
| *TaSBP*-16318-R | tgctcaccatggatccGATCCATATGTCAGAGGTGC |  |
| *TaSBP-*1302-Flag-F | gagagaacacgggggactcttgaccatggtgATGGCCGCAGCGGTGGCTGA | *Noc*Ⅰ |
| *TaSBP-*1302-Flag-R | ttctcctttactagtcagatctaccatggaGATCCATATGTCAGAGGTGC |  |
| *TaSBP-*pYES-F | gcttggtacccgagctcggatccccATGGCCGCAGCGGTGGCTGA | *BamH*Ⅰ |
| *TaSBP-*pYES-R | gctcaccatggtggcgaccggtaccctCTAGATCCATATGTCAGAGG | *Xho*Ⅰ |
| *TaSBP-*110-HA-F | tctagaggatccccgggATGGCCGCAGCGGTGGCTGA | *Sam I* |
| *TaSBP-*110-HA-R | tagagctcggtacccggGATCCATATGTCAGAGGTGC |  |
| *SBP*-qRT-F | AGCACAGGTCTTCTTCCA |  |
| *SBP*-qRT-R | CCAAGTAGAGATAACGGTCAT |  |
| *Ubiquitin-*F | TGACACCATCGACAACGTGA |  |
| *Ubiquitin-*R | GAGGGTGGACTCCTTCTGGA |  |
| *TaSBP*-pGEX-F | gttccgcgtggatccccggaattcATGGCCGCAGCGGTGGCTGA | *EcoR*Ⅰ |
| *TaSBP*-pGEX-R | ccgctcgagtcgacccgggaattcCTAGATCCATATGTCAGAGG |  |
| *ΔTaSBP*-pGEX-F | ggcAGCTCCggcCATGGTGATCCATCCACGAGCCGGCGCTTC |  |
| *ΔTaSBP*-pGEX-R | AGCGTTCCAGCCGGAGTGATGCAGC |  |
